# Object and Scope

**Published:** 1893-11-18

**Authors:** 


### THE STUDY OF MAN.

### I.—OBJECT AND SCOPE.

"The proper study of mankind is man."—Pope.

If the proverbial wanderer from another sphere could be seen summarising his experiences on this planet, we may, without presumption, be very sure that no small space in his note-book would follow the leading title, "Man—What he is, and what he does."

The disinterested observer would naturally regard man, in the first instance, simply as the sole representative of a well-marked division of the order *primates* of the mammalian class of vertebrate animals, allied to the members of its other divisions by well-marked similarities of structure and function, and most nearly to the so-called anthropoid apes, but clearly marked off from them by distinctions of at least generic value. Conspicuous among these are the erect attitude which is normal in man; the relatively enormous development of his cerebral hemispheres, and of the cranium enclosing them; the extreme specialisation of his fore limbs as prehensile organs, and of his hind limbs as means of support and progression; and the rarity of his hairy covering, except on the head, and, in the males of some races, in the neighbourhood of the mouth.

It would be noticed, however, that the species in question, though so manifestly allied to its nearest zoological kindred, presented very strong contrasts in outward appearance, and even in organic structure; and the variations, in particular, in the colour of the skin, the texture of the hair, the proportions of the limbs, and the shape of the skull and its relation to the jaws and other parts of the face, might be held to constitute distinct species or sub-species; though it would be found that all known varieties are fertile with one another, and most of them quite without qualification.

These races are found to be distributed for the most part in distinct geographical areas, and to have been modified to suit the climate and natural products of their habitat. Mixed races, however, occur, and colonies of one race are often found living in the midst of another, in an environment very different from that into which they can be retraced.

The time, mode, and place of man's origin cannot be exactly determined with the palaeontological evidence which is at present available. There are but few undisputed traces of his existence before the close of the last great ice age in the northern hemisphere; and it is possible that his differentiation from the groups of animals which most nearly resemble him, may have been hastened by the marked changes of climate and geographical distribution which took place during that period. It may be added that embryological evidence, and the observation of young children, tends to show that this separation is not of very early date; but that with one or two exceptions the earliest known skulls show no decided inferiority in build or capacity to those of many races which still maintain themselves upon the earth's surface.

Now, if our celestial visitor had concluded his "Anthropology" at the point to which we have just followed him, we should surely have good reason to complain that though from the biological point of view his summary might be adequate and exact, he had, somehow, stopped short of the characters which make man most distinctly human, and fix so great a gulf between him and all other living things. Perhaps we should go on to remark that his detailed craniometry in particular was so much waste ciphering, if he had nothing to say about the manifestations of the mental activity of which those enlarged and elaborately convoluted hemispheres were the physiological seat. And we might indicate to him, in the barest outline, some of the principal directions in which man's mental capacity revealed itself, and how it was this that won for him his unique position in the world as we know it.

All races of men, in the first place, and races of men only, can express by articulate speech to one another something of what passes in the mind of each, so as to communicate not only their feelings and their wants, but also their individual experience of the best means of satisfying them. These modes of speech are very various, but may be grouped according to their structure and rudimentary forms of words. Similarity of language, however, does not prove the speakers to be of kindred blood; for vocabulary, and even general grammatical idioms are readily learned by us from an immigrant people; that language surviving always, which represents the higher stage of culture. It would, perhaps, be needless to point out how much the study of forms of speech may be made to reveal the earliest steps of human progress, no less than of the general principles which govern human thought.

Secondly, man's power of apprehending the relations between himself and his environment has made him pre-eminently the user of means to satisfy his wants; of tools to achieve his ends. He fashions weapons and implements of wood, stone, or metal to secure his living, and to defend himself against attack; builds clothes of skins or textiles, and shelters of wood or stone; and produces fire to cook food, bake pottery, and reduce natural ores—with the result that even the least proficient races have attained a command over the resources of nature such as no other animal at all approaches to.

Similar in kind is the instinct which drives him to share or exchange the products of labour with his fellows, and to secure the benefits of mutual defence and support in orderly, political, and economic societies.

And, lastly, the supreme characteristic of his mental life is a conception of certain ideals of truth, beauty, and moral excellence, never wholly divorced from an impulse in various ways to realise them in the triple glory of science, art, and a good life.

The above sketch will serve to indicate in the barest outline the data and the problems of "the proper study of mankind." For this science, however, which treats of the human species in all its aspects, it is difficult to find a sufficiently comprehensive name. "Anthropology," its commonest title, is ambiguous, because it is often applied, especially by French writers, to the biological department only. "Völkerkunde," its German equivalent, has in the same way become appropriated to ethnology, but has the advantage of falling into line with "Erdkunde," in the parallel group of sciences for which our "physical geography" is a cumbrous and inadequate translation. Perhaps, after all, it will be best to be content with plain English, and call our subject simply "The Study of Man."

